# Rapid and accurate identification and differentiation of *Mycobacterium tuberculosis* and non-tuberculous mycobacteria using PCR kits available in a high-burden setting

**DOI:** 10.3389/fpubh.2024.1358261

**Published:** 2024-04-02

**Authors:** Bernardo Castro-Rodriguez, Greta Franco-Sotomayor, Ángel Sebastián Rodriguez-Pazmiño, Greta Esther Cardenas-Franco, Solón Alberto Orlando, Javier Hermoso de Mendoza, Henry Parra-Vera, Miguel Ángel García-Bereguiain

**Affiliations:** ^1^One Health Research Group, Universidad de Las Américas, Quito, Ecuador; ^2^Instituto Nacional de Investigación en Salud Pública "Leopoldo Izquieta Pérez", Guayaquil, Ecuador; ^3^Universidad Católica Santiago de Guayaquil, Guayaquil, Ecuador; ^4^Universidad Espíritu Santo, Samborondón, Ecuador; ^5^Departamento de Sanidad Animal, Universidad de Extremadura, Cáceres, Spain; ^6^Centro de Investigación Microbiológica (CIM), Guayaquil, Ecuador

**Keywords:** mycobacteria, *Mycobacterium tuberculosis*, atypical mycobacteria, PCR, clinical performance

## Abstract

Infections caused by mycobacteria, including *Mycobacterium tuberculosis* complex (MTBC) and non-tuberculous mycobacteria (NTM), are a major public health issue worldwide. An accurate diagnosis of mycobacterial species is a challenge for surveillance and treatment, particularly in high-burden settings usually associated with low- and middle-income countries. In this study, we analyzed the clinical performance of two commercial PCR kits designed for the identification and differentiation of MTBC and NTM, available in a high-burden setting such as Ecuador. A total of 109 mycobacteria isolates were included in the study, 59 of which were previously characterized as *M. tuberculosis* and the other 59 as NTM. Both kits displayed great clinical performance for the identification of *M. tuberculosis*, with 100% sensitivity. On the other hand, for NTM, one of the kits displayed a good clinical performance with a sensitivity of 94.9% (CI 95%: 89–100%), while the second kit had a reduced sensitivity of 77.1% (CI 95%: 65–89%). In conclusion, one of the kits is a fast and reliable tool for the identification and discrimination of MTBC and NTM from clinical isolates.

## Introduction

There are two main groups of mycobacteria of clinical importance and global distribution: (a) members of the *Mycobacterium tuberculosis* complex (MTBC), which cause tuberculosis; (b) atypical or non-tuberculous mycobacteria (NTM), which are ubiquitous and opportunistic microorganisms that may cause diseases in immunocompromised individuals and exhibit unusual abilities to survive in odd and extreme environmental conditions (1–3); therefore, their distribution appears to be environmentally defined and non-uniform, although it is still poorly defined (4, 5). The group of NTM comprises any mycobacteria other than MTBC or *Mycobacterium leprae*, including over 190 species and subspecies with a wide range of abilities to cause pulmonary and extrapulmonary diseases (6, 7).

In high tuberculosis (TB)-burden countries, the detection and treatment of NTM rarely occur because of poor knowledge about NTM diseases and limited access to laboratory methods required for culture and molecular assays for species level identification. As these locations rely heavily on the microscopy for TB detection, NTM are commonly missed or mistaken for *Mycobacterium tuberculosis* when using acid-fast smears; consequently, patients are inappropriately treated with anti-TB drugs (3, 6, 8). Most countries do not report NTM diseases (3, 4, 6); therefore, the description of burden, trends, and associated risk factors depends on special studies, surveys, and sentinel surveillance programs (5). There has been an increasing trend in the incidence and prevalence of NTM diseases around the world in the last four decades; however, it varies across different regions (3, 5): in the United States, the prevalence of NTM cases increased from 2.4/100,000 in the 1980s to 15.2/100,000 in 2013 (9); Canada reported an increase from 4.9/100,000 in 1998 to 9.08/100,000 in 2010 (10); England, Wales, and Northern Ireland have shown an overall increase from 0.91/100,000 to 7.6/100,000 by 2012 (5); Brazil reported 0.25/100,000 prevalence in 2008 (3, 11); Taiwan reported an increase from 2.65/100,000 in 2000 to 10.17/100,000 in 2008 (12); South Korea shows an increase from 1.2/100,000 in 2003 to 33.3/100,000 in 2016 (5); and in China, the proportion of NTM cases increased from 15.6% in 2013 to 46.1% in 2018 (5); while in sub-Saharan Africa, the prevalence of pulmonary NTM diseases was 7.5% in a period between 1940 and 2016 (13). Additionally, a recent metadata analysis carried out from 2022 to 2024 has shown a continued increase in NTM isolation and disease across regions and in most countries in North America, Europe, and East Asia (14–17). According to one of those reports from 2022, the overall annual rate of change for NTM infection and disease per 100,000 persons/year was 4.0 and 4.1%, respectively (16, 17). Although no prevalence studies for NTM in Ecuador have ever been done, there are some recent case reports of pulmonary plastic surgery-associated infections caused by NTM (18, 19).

Tuberculosis is one of the major causes of death from a single infectious agent in the world and produces an estimate of 10.6 million ill people and 1.3 million deaths worldwide (20). It is a disease that mainly affects vulnerable population groups in countries with low socioeconomic development, worsening existing inequalities. While TB diagnosis and access to treatment improved in recent years in all regions of the world, the effect of the COVID-19 pandemic greatly disrupted the increasing trend of newly diagnosed TB patients and reports worldwide, from 7.1 million in the period 2017–2019 to 5.8 million in 2019–2020; however, an increase was observed to 7.5 million in 2022 (20, 21).

Either TB or mycobacteriosis diagnosis is still a challenge, especially in low- and middle-income countries (3, 20, 22). Bacilloscopy and mycobacterial culture are the gold standards for clinical diagnosis, although they are time-consuming tasks and require a high bacterial load and expertise to manipulate these microorganisms (1, 8, 23). Additionally, those methods do not allow accurate discrimination between MTBC and NTM (3, 6). As antibiotic treatments are totally different for those two groups of mycobacteria (2, 3, 20, 24), several methodologies for discrimination between MTBC or NTM are available, including biochemical tests (2, 3), Sanger sequencing (3, 6), or MALDI-TOF MS (8, 25–27). Furthermore, PCR protocols for the rapid discrimination of MTBC and NTM have been described. Moreover, there are several commercial PCR-based kits that became available recently, such as the Anyplex™ MTB/NTM Real-time Detection V2.0 (Seegene, South Korea) or Advansure™ TB/NTM Real-Time PCR Kit (LG Life Sciences, South Korea), with variable clinical performance depending on the brand and the study (28–32).

The aim of this study was to compare the clinical performance of two commercial PCR kits available in Ecuador for the rapid and accurate identification and differentiation of MTBC and NTM from the culture samples.

## Materials and methods

### Mycobacteria isolates included in the study

A total number of 109 mycobaceteria clinical isolates from the collection of “Centro Nacional de Referencia para Micobacterias” from “Instituto Nacional de Salud Pública e Investigación Leopoldo Izquieta Pérez” (INSPI) were included in the study.

A total of 50 of those cultures were previously characterized as *Mycobacterium tuberculosis* following the Pan-American Health Organization guidelines using the Kudoh – Ogawa method (33–35) and also characterized by 24 Mycobacterial Interspersed Repetitive-Unit Variable Number of Tandem Repeats (MIRU-VNTR) (36, 37).

The other 59 cultures corresponded to NTM isolated from skin purulent lesions using the Kudoh Ogawa method. Those cultures were characterized by matrix-assisted laser desorption/ionization-time of flight mass spectrometry (MALDI-TOF MS), as described elsewhere (27, 38), and included 29 *Mycobacterium abscessus* isolates, 9 *Mycobacterium farcinogenes* isolates and 19 *Mycobacterium fortuitum* isolates, 1 *Mycobacterium parafortuitum* and 1 *Mycobacterium novocastrense* isolates. Those NTM isolates are the most frequent within the area of study in Ecuador and are commonly associated with infections caused by plastic surgery (personal communication from clinical laboratories to the authors).

### Mycobacteria culture heat inactivation and DNA isolation

Mycobacterial colonies were harvested from cultures, resuspended in Tris-EDTA (TE) buffer (10 mM Tris–HCl, 1 mM EDTA, pH 8.0), and then inactivated at 95°C for 15 min. After heat inactivation, all samples were centrifuged for 5 min at 10,000 *g*, and the supernatant was directly used for molecular procedures, as reported elsewhere (39–42). The heat inactivation process was performed within the BSL2+ facility of the “Centro Nacional de Referencia para Micobacterias” from INSPI to prevent occupational exposure as it has been recommended (19).

### qPCR using the VIASURE real-time PCR detection kit – MTBC + NTM (CerTest BIOTEC, Zaragoza, Spain) (“Viasure kit”)

This IVD-CE marked qualitative real-time PCR assay is directed to the detection and differentiation of *Mycobacterium* genus, the MTBC, and/or the specific differentiation of modern *M. tuberculosis* species (L2, L3, and L4 families) DNA from clinical strains and lower-track respiratory samples. The process is PCR-based with the use of specific fluorescent probes and primers for the amplification of conserved regions: a segment of the 16S rRNA gene for the detection of *Mycobacterium* representatives, insertion sequences IS*6110* and IS*1081* for the identification of mycobacteria that belong to the MTBC, and/or a fragment of the TbD1 deletion region, which allows the specific detection of *M. tuberculosis* strains classified as modern strains of the L2, L3, and L4 families. The format of the VIASURE RT-PCR Detection Kit used in this study contains 12 × 8-well strips, in which all reagents are lyophilized in each well for the RT-PCR assay: specific primers and probes, dNTPs, buffer, and polymerase, together with an Internal Control to discard inhibition of the polymerase activity. According to the manufacturer, after the addition of 15 μL of rehydration buffer to each well, a volume of 5 μL of the DNA sample was added to select wells, then 5 μL of reconstituted MTBC + NTM-positive control and 5 μL of MTB/NTM-negative control were added to separate wells for a final reaction volume of 20 μL in each well. A thermocycler (CFX96 from Bio-Rad) was programmed to detect signals (cycle threshold (Ct) ≤40) in the ROX channel (for 16S rRNA gene – *Mycobacterium* species), FAM channel (for IS*6110* and IS*1081* sequences – MTBC members), Cy5 channel (for TbD1 deletion region – “modern” *M. tuberculosis*), and HEX channel (for Internal Control), using the following PCR conditions: one cycle of 95°C for 2 min, followed by 45 cycles of 95°C for 10 s and 60°C for 50 s; fluorogenic data were collected after each cycle of this step (43).

### qPCR using the *Mycobacterium* multiplex nucleic acid diagnostic kit (multiplex PCR – fluorescence probing) (Sansure Biotech, Changsha, People’s Republic of China) (“Sansure kit”)

This commercial PCR kit is designed to detect DNA from seven types of common clinical mycobacteria: *M. tuberculosis* (MTB), *Mycobacterium kansasii* (MK), *Mycobacterium avium* (MA), MF, *Mycobacterium abscessus* subsp. *abscessus* (MAA), *Mycobacterium abscessus* subsp. *massiliense* (MAM), and *Mycobacterium intracellulare* (MI). The detection is based on two kinds of PCR-technical principles: the first uses specific fluorescent probes to detect one target, and the second identifies another target by melting curve analysis, thus achieving simultaneous detection of both targets using the same fluorescence channel. The *Mycobacterium* Multiplex Nucleic Acid Diagnostic Kit (Multiplex PCR – Fluorescence Probing) has an open format; therefore, the volume of added reagents can be modified while maintaining the concentration of the reaction established by the manufacturer: each tube contained 13.28 μL of the MTB/NTM-PCR Mix, 0.23 μL of the MTB/NTM Enzyme Mix, and 1.5 μL of DNA from samples, while 1.5 μL of the MTB/NTM-positive control and 1.5 μL of the MTB/NTM-negative control were added to the reaction mixture in different tubes for a final reaction volume of 15 μL in each tube. The thermocycler was programmed as follows: one cycle of 50°C for 2 min; one cycle of 94°C for 3 min; and 45 cycles of 94°C for 10 s, 60°C for 20 s, and 75°C for 20 s (fluorogenic data were collected at the end of each cycle of this step); then, one cycle of continuous increase in temperature from 62°C to 75°C for fluorescence data collection for the melting curve analysis. The signal of specific fluorescent probes (Ct ≤ 39) is detected in the ROX channel for MF, in the FAM channel for MK, in the Cy5 channel for Internal Control, and in the HEX channel for MA, while DNA of MI is detected by the presence of a melting curve in the ROX channel (melting temperature peak (Tm peak): 70.5 ± 1°C), MAA is detected in the FAM channel (Tm peak: 69.5 ± 1°C), MAM is detected in the Cy5 channel (Tm peak: 68 ± 1°C), and MTB is detected in the HEX channel (Tm peak: 67 ± 1°C) (44).

### Sanger sequencing for identification of NTM species

By means of Sanger sequencing and BLAST searches, NTM culture strains were evaluated for identification using the molecular markers 16S, *rpoB*, and *hsp65* genes. These three markers have been widely used to identify mycobacteria (45–49). The PCR amplification of the three genes was performed using GoTaq^®^ Green Master Mix (Promega, Wisconsin, United States) following protocols described elsewhere (46, 47, 49). Amplification of fragments was confirmed by electrophoresis in 2% UltraPure™ Agarose (Invitrogen, California, United States) gels of 15 cm x 10 cm in 0.5X Tris-boric acid-EDTA buffer at 100 V for 3 h using a ladder 100 bp Plus Opti-DNA Marker (Cat. No.: G016, Applied Biological Materials Inc., British Columbia, Canada) for size determination. Amplicons were sequenced with the Sanger method and analyzed with the ABI 3500xL Genetic Analyzer from Applied Biosystems at the Service Department of Universidad de las Americas, Quito, Ecuador. The obtained sequences were curated using Geneious^®^ v. 11.0.4 (Dotmatics, United Kingdom) and then proceeded to their identification by nucleotide-BLAST (NCBI, United States) search using default parameters.

### Ethics statement

The access to this micobacteria collection was approved by IRB from Universidad de Las Américas (code 2024-EXC-001). All samples were anonymized, and no personal data of the patients were made available.

## Results

### Identification of MTBC cultures using the two commercial qPCR kits included in the study

The 50 MTBC cultures yielded a positive result for MTBC DNA detection either with the Viasure or the Sansure kit, showing a sensitivity of 100% (Tables 1, 2; Supplementary Data 1).

**Table 1:** Clinical performance of the Viasure MTBC+NTM RT-PCR kit for the detection of the *Mycobacterium tuberculosis* complex.

		Reference method		Total
		Positive	Negative	
Viasure PCR kit	Positive	50	0	50
	Negative	0	0	0

**Table 2:** Clinical performance of Sansure *Mycobacterium* Multiplex RT-PCR kit for detecting *Mycobacterium tuberculosis* complex.

		Reference method		Total
		Positive	Negative	
Sansure PCR kit	Positive	50	0	50
	Negative	0	0	0

### Identification of NTM using the VIASURE real-time PCR detection kit – MTBC + NTM

Fifty-six out of the fifty-nine NTM cultures previously characterized by MALDI-TOF MS were positive for *Mycobacterium* genus for the Viasure kit according to the manufacturer’s instructions (Ct ≤ 40 in the ROX channel). The remaining three cultures were identified as MTBC cultures by the Viasure kit (Ct ≤ 40 simultaneously for ROX and FAM channels), which means a sensitivity of 94.9% (CI 95%: 89-100%) for detection of NTM (Table 3; Supplementary Data 1).

**Table 3:** Clinical performance of the Viasure MTBC+NTM RT-PCR kit for the detection of non-tuberculous Mycobacteria.

		Reference method		Total
		Positive	Negative	
Viasure PCR kit	Positive	56	0	56
	Negative	3	0	3

Those three NTM cultures identified as MTBC by the Viasure kit were subjected to Sanger sequencing to confirm the MALDI-TOF MS result. Two strains were identified as NTM through the *rpoB* and 16S markers, while one of the strains could not be further characterized by Sanger sequencing.

Additionally, the specificity of the Viasure kit was 94.9% (CI 95%: 89–100%) and 100% for MTBC and NTM culture identification, respectively.

### Identification of NTM using the *Mycobacterium* multiplex nucleic acid diagnostic kit (multiplex PCR – fluorescence probing)

Thirty-seven out of the fifty-nine NTM cultures previously characterized by MALDI-TOF MS were positive for *Mycobacteria* for the Sansure kit, according to the manufacturer’s instructions (see Methods). The remaining 19 NTM cultures were identified as negative samples, either for MTBC or NTM, which means a sensitivity of 62.7% (CI 95%: 50–75%) for the detection of NTM (Table 4; Supplementary Data 1).

**Table 4:** Clinical performance of Sansure *Mycobacterium* Multiplex RT-PCR kit for detection of non-tuberculous mycobacteria.

		Reference method		Total
		Positive	Negative	
Sansure PCR kit	Positive	37	0	37
	Negative	22	0	22

As the Sansure kit is designed for the identification of certain *Mycobacteria* species, not including *M. farcinogenes*. *M.parafortuitum* and *M.novocastrense*, we considered the exclusion of the eleven culture samples of that species for clinical performance analysis in Table 5. For the remaining forty-eight mycobacteria culture, thirty-seven were detected by Sansure kit, as mentioned above, which means that sensitivity of the Sansure kit for the detection of the NTM species indicated by the manufacturer was 77.1% (CI 95%: 65–89%).

**Table 5:** Clinical performance of Sansure *Mycobacterium* Multiplex RT-PCR kit for detection of non-tuberculous mycobacteria, including only these species: *M. kansasii, M. abscessus, M. abscessus* subsp. *massiliense, M. avium, M. fortuitum,* and *M. intracellulare*.

		Reference method		Total
		Positive	Negative	
Viasure NTM	Positive	37	0	37
	Negative	11	0	11

Additionally, the specificity of the Sansure kit was 100% for either MTBC or NTM culture identification.

### Reproducibility analysis

The reproducibility of both commercial PCR kits was assessed by running in triplicate all the non-matching samples compared to the standard method, as well as 20% of the matching results. We found 100% reproducibility for all samples analyzed for both the Sansure and Viasure PCR kits.

## Discussion

Mycobacterial infections are a major public health issue worldwide due to different conditions related to these microorganisms (i.e., virulence, sensitivity, and resistance to antibiotics), the host (i.e., comorbidities and immunological status), and the environment where they develop (i.e., precarious living conditions, socio-economic inequity, and human migration) (40, 50, 51). Furthermore, the infrastructure, skills, and time needed to perform the microbiological procedures required for sampling, culturing, and identifying mycobacteria are negatively impacting its diagnosis (3, 4, 6). Additionally, proper identification of mycobacteria is fundamental for accurate treatment, as not only the antibiotic therapy varies between TB and mycobacteriosis but also depends on the NTM species (2, 3).

Therefore, fast and reliable identification and differentiation of infectious mycobacteria has become a critical priority in the field of microbiological diagnosis, and the implementation of PCR-based techniques has allowed the development of a variety of commercially available kits that perform identification assays in a matter of hours (52–54). For instance, there are several reports addressing the clinical performance of some commercial kits, such as the Anyplex™ MTB/NTM Real-time Detection V2.0 (Seegene, South Korea), with reported sensitivities for MTB detection ranging from 71 to 86% and reported specificities ranging from 94.9 to 99%; while for NTM detection, the reported sensitivities ranged between 44.9 and 100% and the reported specificities ranged from 97.7 to 97% (29, 31, 32, 55). For the Advansure™ TB/NTM Real-Time PCR Kit (LG Life Sciences, South Korea), MTB detection sensitivity ranges between 78.1 and 96.2 and specificity ranges from 93.8 to 96.2%, while NTM detection sensitivity was in the range of 25–51.7% and specificity ranges between 97.8% and 98.3% (31, 32). All these cited reports worked with DNA extracted from clinical samples for the PCR-based assay and compared against culture results, which is considered the gold standard for mycobacterial diagnosis (29, 33). Although these reports endorse the use of PCR-based methods as suitable techniques for rapid mycobacterial detection, clinical performance evaluation studies are necessary as a wide range of sensitivity values were obtained, not only depending on the commercial brand but especially when comparing MTBC with NTM. To date, those reports support that, at least for NTM identification, PCR testing should be accompanied by microbiological analysis for a definitive diagnosis (29, 52, 54).

In this study, to the best of our knowledge, we present the first clinical performance evaluation of two commercial PCR kits recently available for MTBC and NTM identification. For rapid identification of MTBC cultures, both Viasure and Sansure kits are highly reliable tools with 100% sensitivity compared to gold standard methods. However, there was a slight reduction in MTBC identification specificity for the Viasure kit, although co-infection of MTBC and NTM not detected by MALDI-TOF MS cannot be totally ruled out.

By contrast, we found strong differences in the clinical performance of the Viasure and Sansure kits for the identification of NTM. The Viasure kit exhibited a great clinical performance with 94.9% (CI 95%: 89–100%) sensitivity and 100% specificity. However, even for the set of NTM species that the Sansure kit was designed for, the sensitivity value obtained was 77.1% (CI 95%: 65–89%). The importance of conducting these kinds of studies in different geographical settings, as the distribution of NTM species varies worldwide, has been emphasized. For instance, the NTM species included in this study are some of the most common in skin lessons associated with plastic surgery in Ecuador (personal communication to the authors). However, one of the kits evaluated in the study is not designed for the detection of three of those species, not within the most prevalent NTM species in other settings.

Considering that both commercial kits are also recommended for the direct detection of mycobacteria in clinical samples, we would anticipate poor clinical performance for the Sansure kit for NTM detection in clinical samples, while further experiments will be needed to address the clinical performance of the Viasure kit. This poor performance is actually and simultaneously the main limitation and further direction of our study. We are currently working in a study to address the clinical performance of these two PCR commercial kits for the detection of MTBC and NTM directly in clinical samples from TB and mycobacteriosis patients.

In conclusion, one of the PCR kits evaluated in this study (Viasure kit) is a fast and accurate tool for the identification and differentiation of MTBC and NTM from clinical isolates. This type of molecular test could help with the fast and affordable triage of clinical isolates in middle- to high-burden settings such as Ecuador, where differential diagnosis for TB and mycobacteriosis is a challenge.

## Data availability statement

The original contributions presented in the study are included in the article/supplementary material, further inquiries can be directed to the corresponding author.

## Author contributions

BC-R: Data curation, Formal analysis, Investigation, Methodology, Project administration, Writing – original draft, Writing – review & editing. GF-S: Data curation, Formal analysis, Funding acquisition, Investigation, Supervision, Methodology. AR: Data curation, Methodology, Writing – review & editing. GC-F: Writing – review & editing, Investigation, Methodology. SO: Supervision, Validation, Writing – review & editing, Investigation. JH: Supervision, Validation, Writing – review & editing, Resources. HP-V: Conceptualization, Funding acquisition, Resources, Supervision, Validation, Writing – review & editing. MG-B: Conceptualization, Data curation, Formal analysis, Funding acquisition, Investigation, Supervision, Validation, Writing – original draft, Writing – review & editing.
